# Brain–computer interfaces in post-stroke rehabilitation: a neurotechnological leap toward functional recovery

**DOI:** 10.1097/MS9.0000000000003950

**Published:** 2025-09-26

**Authors:** Eisha Ali, Sheza Kamran, Asad Ali Ahmed Cheema

**Affiliations:** a Department of Medicine, FMH College of Medicine and Dentistry, Lahore, Pakistan; b Department of Medicine, International School of Medicine, International University of Kyrgyzstan, Bishkek, Kyrgyzstan

*To the Editor,*

This manuscript has been prepared in compliance with the TITAN Guidelines 2025 for transparency, integrity, and appropriate use of artificial intelligence in scholarly publishing^[1]^.

Brain-Computer Interfaces (BCIs) are a promising adjunct in post-stroke rehabilitation, especially for patients with persistent motor deficits due to recent advancements in neurotechnology. Even in situations where conventional physiotherapy produces little improvement, BCIs provide a novel way to re-engage neuroplastic mechanisms by converting cortical signals into actionable outputs. A BCI intervention dramatically enhanced upper limb motor function and altered important brain networks, such as the visual and dorsal attention networks, according to a randomized controlled trial by Ma *et al* suggesting that they can actively support neuroplasticity and functional recovery in stroke patients^[2]^.

Recent research shows that by strengthening volitional intent, BCI-mediated feedback whether through virtual reality, robotic exoskeletons, or functional electrical stimulation, can improve motor relearning. Research conducted by Zhang *et al*. in stroke patients, demonstrated that exoskeleton-guided passive movement produced standardized EEG patterns, enhancing proprioceptive-driven cortical engagement and the generalizability of BCI decoding algorithms^[3]^. In a similar way, Nguyen et al. created a VR–BCI system that converted EEG signals into immersive rehabilitation exercises, showing enhanced sensorimotor feedback that improved motor recovery and engagement^[4]^. There are still several translational issues, and additional standardization is required due to the variations in neural signal quality, patient adherence, and long-term retention of motor gains. Furthermore, as BCIs advance from research to clinical implementation, ethical issues pertaining to cognitive load, data privacy, and equitable access must be addressed.

Although BCIs are not yet widely used, their development points to a paradigm shift in the way we view stroke recovery. Hybrid BCI systems that incorporate adaptive feedback loops, AI-driven signal decoding, and customized rehabilitation protocols ought to be the focus of future research. In their review of recent developments in machine learning-based BCI systems, Elashmawi *et al* emphasized how intelligent signal processing and user-specific adaptation could improve motor recovery^[5]^. These systems have the potential to maximize therapeutic effectiveness while reducing user fatigue, which is a significant obstacle in long-term neurorehabilitation.

Future research in this area will determine whether BCIs can improve outcomes by enabling neuroplastic restoration through the integration of AI and adaptive feedback. However, clinical success hinges on resolving issues with long-term usability, ethical concerns, and technical variability.

## Data Availability

Not applicable – no new data were generated or analyzed in this letter to the editor.

## Research registration unique identifying number (UIN)

Not applicable – This letter to the editor does not involve human participants and is based on previously published literature.
